# Medical Department of the Army

**Published:** 1850-07

**Authors:** 


MEDICAL DEPARTMENT OF THE ARMY.

By a board of Army surgeons which was convened on the 15th ult., in the city of New York, for the examination of assistant surgeons for promotion, and of applicants for appointment to the Medical Staff of the Army, Assistant Surgeons Charles H. Laub, Richard H. Coolidge, and Alexander S. Witherspoon were examined and found qualified for promotion.

The following applicants for the appointment of Assistant Surgeon in the Army were also examined and approved :—

Samuel, W. Crawford, Pennsylvania; William H. Tingley, Pennsylvania; John J. Milhau, New York; Aquila T. Ridgley, Louisiana; Charles Page, Virginia; Archibald Taylor, Virginia; Charles Sutherland, Pennsylvania.

